# The effects of auditory enrichment on zebrafish behavior and physiology

**DOI:** 10.7717/peerj.5162

**Published:** 2018-07-23

**Authors:** Heloísa H. A. Barcellos, Gessi Koakoski, Fabiele Chaulet, Karina S. Kirsten, Luiz C. Kreutz, Allan V. Kalueff, Leonardo J. G. Barcellos

**Affiliations:** 1 Programa de Pós-Graduação em Farmacologia, Universidade Federal de Santa Maria (UFSM), Santa Maria, Rio Grande do Sul, Brazil; 2 Faculdade de Agronomia e Medicina Veterinária, Universidade de Passo Fundo, Passo Fundo, Rio Grande do Sul, Brazil; 3 Programa de Pós-Graduação em Bioexperimentação, Universidade de Passo Fundo (UPF), Passo Fundo, Rio Grande do Sul, Brazil; 4 Programa de Pós-Graduação em Ciências Ambientais, Universidade de Passo Fundo, Passo Fundo, Rio Grande do Sul, Brazil; 5 School of Pharmacy, Chongqing University, Chongqing, China; 6 Institute of Translational Biomedicine, St. Petersburg State University, Saint Petersburg, Russia; 7 The International Zebrafish Neuroscience Research Consortium (ZNRC), Slidell, LA, USA; 8 Research Institute for Marine Drugs and Nutrition, Guangdong Ocean University (GDOU), Guangdong, China; 9 Ural Federal University, Ekaterinburg, Russia; 10 ZENEREI Research Center, Slidell, LA, USA; 11 Institute of Experimental Medicine, Almazov National Medical Research Center, St. Petersburg, Russia; 12 Russian Research Center for Radiology and Surgical Technologies, Pesochny, Russia; 13 Research Institute of Physiology and Basic Medicine, Novosibirsk, Russia

**Keywords:** Auditory enrichment, Fish welfare, Stress, Anxiety, Immune genes, Exploratory behavior

## Abstract

Environmental enrichment is widely used to improve welfare and behavioral performance of animal species. It ensures housing of laboratory animals in environments with space and complexity that enable the expression of their normal behavioral repertoire. Auditory enrichment by exposure to classical music decreases abnormal behaviors and endocrine stress responses in humans, non-humans primates, and rodents. However, little is known about the role of auditory enrichment in laboratory zebrafish. Given the growing importance of zebrafish for neuroscience research, such studies become critical. To examine whether auditory enrichment by classical music can affect fish behavior and physiology, we exposed adult zebrafish to 2 h of Vivaldi’s music (65–75 dB) twice daily, for 15 days. Overall, zebrafish exposed to such auditory stimuli were less anxious in the novel tank test and less active, calmer in the light-dark test, also affecting zebrafish physiological (immune) biomarkers, decreasing peripheral levels of pro-inflammatory cytokines and increasing the activity of some CNS genes, without overt effects on whole-body cortisol levels. In summary, we report that twice-daily exposure to continuous musical sounds may provide benefits over the ongoing 50–55 dB background noise of equipment in the laboratory setting. Overall, our results support utilizing auditory enrichment in laboratory zebrafish to reduce stress and improve welfare in this experimental aquatic organism.

## Introduction

Numerous studies consistently show benefits of music, especially classical music, to humans (Binns-Turner et al., 2011; Smolen, Topp & Singer, 2002; Villarreal et al., 2012; Cervellin & Lippi, 2011). For example, classical music increases human wellbeing, reduces stress, and anxiety, as well as normalizes blood pressure, immune function, and cognitive performance (Rickard, Toukhsati & Field, 2005). Musical “auditory” environmental enrichment can be also used to improve welfare of laboratory animals, with clear positive behavioral effects and overall stress relief reported in multiple species, including dogs, primates, pigs, horses, and rodents (Alworth & Buerkle, 2013). In contrast, uncontrollable chronic noise exposure in the laboratories may impair welfare of the experimental animals (Patterson-Kane & Farnworth, 2006), and therefore represents a detrimental factor in neurobehavioral studies (also see Kettelkamp-Ladd, 1993).

Like mammals, fish have a well-developed auditory system (Fay & Popper, 2000). Fish perceive various sounds within aquatic environment, demonstrating selectivity for music tempo (Catli, Yildirim & Turker, 2015) and discriminating sound intensity, frequency, and the source location (Fay & Popper, 2000). Fish hearing involves otolith organs (saccule, lagena, and utricle), and their “auditory filters” operate in the range <40 Hz to >1 KHz, depending of the species (Fay & Popper, 2000).

Despite the negative effects of noise on many fish species (Vazzana et al., 2017; Celi et al., 2016; Buscaino et al., 2010; Filiciotto et al., 2014), classical music exposure accelerates reproduction in several fish species (Papoutsoglou et al., 2007; Papoutsoglou et al., 2010; Imanpoor, Enayat Gholampour & Zolfaghari, 2011; Catli, Yildirim & Turker, 2015) by positively modulating their physiological and metabolic states (Papoutsoglou et al., 2010). The reaction of fish to music has also been examined in some earlier studies. For example, exposed to classical music in culture ponds, carps (Papoutsoglou et al., 2007), and turbots (Catli, Yildirim & Turker, 2015) grew larger and fed more efficiently. In addition, fish are capable of hearing sounds from the aquatic ambient (Popper & Fay, 2011). However, there are scarce in-depth systematic studies of potential effects of environmental music exposure on behavioral and physiological biomarkers in fishes and of the impact of aquatic research and housing laboratory environments on such fish phenotypes.

The zebrafish *(Danio rerio)* is a widely used animal model organism in neuroscience research (Papoutsoglou et al., 2007; Sicca et al., 2016; Levitas-Djerbi & Appelbaum, 2017; Uchiyama et al., 2012; Kalueff et al., 2013). They are genetically and physiologically similar to others vertebrates, such as rodents and humans (Howe et al., 2013), and possess a well-described behavioral repertoire (Kalueff et al., 2013) and stress neuroendocrine axis (Stewart et al., 2014; Kalueff, Stewart & Gerlai, 2014; Alsop & Vijayan, 2009). In zebrafish, environmental enrichment research is only beginning to emerge. For example, enrichment using other sensory modalities is known to blunt zebrafish stress responses and improve welfare (Schroeder et al., 2014; Collymore, Tolwani & Rasmussen, 2015; Manuel et al., 2015; Giacomini et al., 2016). However, little is known about the impact of sound exposure, and its potential as an auditory enrichment, on zebrafish behavior and physiology. In addition to raising a scientific interest, this question also becomes important practically since zebrafish research facilities routinely utilize aquatic systems with circulating water and/or stationary tanks with aerators and water filters, each generating significant background noise. Although critical from an animal welfare and data reproducibility standpoints, these aspects have not been systematically assessed in zebrafish laboratories. Likewise, despite the well-known positive effects of musical environmental enrichment in rodents and other species, there are no studies assessing the effects of music on zebrafish behavior and physiology. To address this knowledge gap, here we examine the effects of auditory environmental enrichment via chronic classical music exposure on zebrafish behavioral and physiological responses. Specifically, we wanted to assess how repeated exposure to such auditory enrichment can modulate zebrafish stress/anxiety-related behavior in two different behavioral models, fish endocrine (cortisol) and physiological (immune) responses as well as the expression of selected CNS genes, compared to the control group of fish unexposed to auditory enrichment.

## Materials and Methods

### Animals

A total of 36 mixed-sex (1:1 female:male ratio) adult one-year old wild-type short-fin outbred zebrafish were used in this study. Fish were bought from a local commercial supplier (Recanto dos Peixes, Marau, Brazil) and were acclimated to the University of Passo Fundo animal facility for six months prior to testing. The animals were housed for 20 days in the UPF aquatic laboratory facility (including a five-day acclimation and a 15-day testing). The fish were kept, in groups of three, in 12 3-L glass tanks (20 height × 15 depth × 14 width cm), under constant aeration and a 14 h L: 10 h D cycle. Water temperature was maintained at 27.5 ± 1.3 °C, with pH 7.7 ± 0.08, dissolved oxygen at 5.6 ± 0.5 mg/L and ionized ammonia <0.022 ppm. Water was partially (30%) changed every two days throughout the entire experimentation period. Relevant to the goals of this study, the baseline noise levels in the laboratory were 50–55 dB (with frequency varying from 240 to 420 Hz), and mostly consisted of sounds produced by fish husbandry equipment, such as aerators and water pumps. Control fish were kept away from the room used for music exposure of the experimental (“enriched”) cohort. No other sounds were presented to the control group, and their only difference from the experimental group was the lack of music exposure during the experiments.

### Ethical note

All experimental procedures were performed in accordance with the guidelines of the National Council of Animal Experimentations Control (CONCEA) of Brazil. This study was approved by the Ethics Committee for Animal Use of the University of Passo Fundo, Brazil (UPF protocol 040/2017).

### Experimental procedures

Our study aimed to assess zebrafish behavioral and endocrine (cortisol) responses and the expression of selected immune and hypothalamus-pituitary-interrenal axis-related genes in the brain. Behavioral testing utilized the novel tank (NTT) and the light-dark test (LDT) tasks following a 15-day repeated exposure to music. For this, fish were divided into two groups kept in six glass tanks (three fish per tank, *n* = 18 per group). One group was subjected for 15 days to two sessions of 2-h selection of Vivaldi’s music (Table 1), chosen here as the representative “Popular collection.” The intensity level of the music was arbitrarily set at 65–75 dB (with frequency varying from 330 to 506 Hz), based on considerations of safety and overall pleasantness of sounds for human ears (Brookhouser, 1994). Music and background noise intensities and frequencies in this study were assessed outside the water using the Sound Level Meter Application (available online from Google Play at https://play.google.com/store/apps/details?id=com.bolshakovdenis.soundanalyzer) on a Samsung Galaxy S6 smartphone (Samsung Brazil, Brasília, Brazil, 2017). The morning daily session started at 8:30 am, followed by the second (afternoon) daily exposures at 17:00 pm. All fish were fed twice a day, 30 min prior to each the music exposure sessions, to mitigate the effect of hunger on their behavior. On the final day, fish were fed at 8:00 am and submitted to behavioral assays (NTT or LDB test, *n* = 10–12 per group each) at 10:30 am. After testing in either assay for 6 min, the fish were individually removed by the net and immediately euthanized with ice-cold water, decapitated and stored at liquid nitrogen for 30 s. The 6-min behavioral testing used here in both assays is a standard, commonly used testing protocol in zebrafish neurobehavioral analyses (Egan et al., 2009). Their trunks were then stored at −8 °C for cortisol analyses, and their heads stored at −80 °C for RNA and DNA extraction and analyses of the genes expression using the real-time PCR (Table 2). The control group underwent the same housing, handling, and testing procedures, but was unexposed to music throughout the study. The selection of “no-music” control (rather than exposing controls to other types of music or noise) for our study was based on the specific research question we aimed to address. The main focus of our study was to examine the potential of music exposure as an environmental enrichment. Respectively, for the stated experimental design, the selection of Vivaldi (vs. other composer) was not critical, serving as an example of a mild relaxing music frequently used in auditory enrichment studies in other species (Rickard, Toukhsati & Field, 2005; Papoutsoglou et al., 2007). Because we wanted to assess whether repeated exposure to music in general can affect fish physiology and behavior, only direct comparison of music-exposed vs. unexposed fish groups was appropriate. Albeit interesting and clearly meriting further scrutiny, comparing Vivaldi’s music with other music or sounds was beyond the scope of the present study.

**Table 1:** Summary of Vivaldi’s music classical collection utilized in the present study.

Concert	Music
In C major	Allegro molto 5.18
	Larghetto 3.10
	Allegro 1.35
N.1 “Spring”	Allegro 3.29
	Largo 2.54
	Danza pastorale: Allegro 4.26
For mandolin, strings, and basso continuo no.1	Allegro 2.56
	Largo 3.0
	Allegro 3.03
For two violin, strings, and harpsichord	Allegro 3.09
	Andante 2.46
	Allegro 2.43
For two oboes, bassoon, two horns, violin, strings, and organ	Allegro 4.26
	Largo 1.32
	Allegro 4.05
N.10	Allegro 4.13
	Largo, Larghetto 3.20
	Allegro 3.29

**Table 2:** The qPCR primers used in the present study.

Gene	Primer (5′–3′)	Efficiency (%)	Accession number
StAr	F: CCTGTTTTCTGGCTGGGATG R: GGGTCCATTCTCAGCCCTTAC	101	NM_131663.1
POMC	F: CGCAGACCCATCAAGGTGTA R: CGTTTCGGCGGATTCCT		AY125332.2
CRF	F: ACGCACAGATTCTCCTCGCC R: TCCGCGGCTGGCTGATT		NM001007379.1
cFOS	F: CAGCTCCACCACAGTGAAGA R: GCTCCAGGTCAGTGTTAGCC	97	DQ003339.2
BGR	F: ACAGCTTCTTCCAGCCTCAG R: CCGGTGTTCTCCTGTTTGAT		DQ017615.1
BDNF	F: CGCCGTTACTCTTTCTCTTGG R: CCATTAGTCACGGGGACCTTC	102	NM_001308648.1
*β-2-microglobulin*	F: GCCTTCACCCCAGAGAAAGG R: CGGTTGGGATTTACATGTTG		NM_131163.2
TNF-α	F: GACCACAGCACTTCTACCG R: ACATTTTCCTCACTTTCGTTCAC		NM_212859
IL-1β	F: GCTGGAGATGTGGACTTC R: ACTCTGTGGATTGGGGTTTG	100	NM_212844
INF-γ	F: TGCCTCAAAATGGTGCTACTC R: AATCGGGTTCTCGCTCCTG		AB158361.1
IL-4	F: TCTCTGCCAAGCAGGAATG R: CAGTTTCCAGTCCCGGTATATG		AM403245.2
IL-12	F: CTGTAGGATCCATCCAAACATCT R: CACTGGCACTTCTACCCTATTT		AB183002.1
IL-10	F: CTCTGCTCACGCTTCTTCTT R: GCTCCCTCAGTCTTAAAGGAAA		BC163038.1
β-Actin	F: GCAAAGGGAGGTAGTTGTCTAA R: GAGGAGGGCAAAGTGGTAAA	99	AF057040.1

#### The novel tank test

The novel tank test was a rectangular glass tank (24 width × 8 depth × 20 high cm), as described previously (Mocelin et al., 2015). Fish were video-recorded for 6 min by a Logitech Quickcam PRO 9000 camera located in front of the tank, and their videos were then analyzed offline by automated ANY-maze^®^ software, assessing time spent in top, middle, and bottom zones (s), number of bottom entries, distance traveled in each zone (m), absolute turn angle in each zone (°), total time spent in mobility (s), according to the Zebrafish Neurobehavioral Catalog (Kalueff et al., 2013).

#### The light-dark test

The LDT was a rectangular apparatus (45 width × 10 depth × 15 high cm), with a five-cm central area separated by two sliding doors (Magno et al., 2015). The apparatus was filled with a five-cm deep water, and fish were individually introduced into the central chamber for 30 s for acclimation. The partition was then raised one cm above the tank floor, to allow zebrafish to swim freely between the sides of the apparatus. Fish were filmed for 6 min and their videos were then analyzed offline using ANY-maze^®^ software, assessing the light zone rotations (complete 360^o^ circling), distance traveled (m), mean speed (m/s), and time spent in zone (s).

#### Cortisol extraction and measurement

The procedure was performed according to (Sink, Lochmann & Fecteau, 2008) using body trunk samples previously stored at −8 °C. Cortisol levels were determined by enzyme-linked immune sorbent assay kit (EIAgen CORTISOL test; BioChem ImmunoSystems, Rome, Italy) from tissue extracts re-suspended in PBS buffer (Oliveira et al., 2014). The accuracy was tested by calculating the recoveries from samples spiked with known amounts of cortisol (50, 25, and 12.5 ng/mL), the mean detection of spiked samples was 94.3%. All cortisol values were adjusted for recovery with the following equation.

$${\rm{Cortisol}}\;{\rm{value}} = {\rm{Measured\;value}} - 1.0604.$$

#### RNA extraction, cDNA synthesis, and gene expression analysis

The brains of three fish per sample were pooled (total *n* = 6 samples per an 18-fish group) and used for RNA extraction. The protocol consisted of tissue lysis using the Tissuelyser LT^®^ (Qiagen, Hilden, Germany), RNA extraction using RNeasy^®^ Mini Kit (Qiagen, Hilden, Germany), and DNAse I amplification grade treatment (Invitrogen, Carlsbad, CA, USA) to eliminate genomic DNA. The RNA quality and concentration was measured by spectrophotometry (Nanophotometer Pearl^®^; IMPLEN, Munich, Germany). For cDNA synthesis, one μg of total RNA was used for the reverse transcription assay, using QuantiTect^®^ III Reverse Transcription kit (Qiagen, Hilden, Germany). The real time PCR (qPCR) was performed using Rotor-Gene Q equipment (Qiagen, Hilden, Germany) with initial denaturing at 95 °C for 10 min followed by 40 cycles of 95 °C for 30 s, 60 °C for 30 s, and 72 °C for 30 s. At the end, a standard melting curve was included to confirm the specificity of the amplified product. The amplification of the mRNA of the selected genes (Table 2) was compared to β-actin, used as a housekeeping gene. For the calibration curve, each gene was cloned and transformed into competent One Shot TOP10 *E. coli* and cultured in LB supplemented with ampicillin. The cloning was confirmed by PCR and the resulting plasmid was extracted. Then, the calibration curve consisted of decimal dilutions (1:10) of each cloned gene. To compare the results from different groups, the same threshold value (0.10) was used. The relative quantification of gene expression was performed using the 2^−ΔΔct^ formula (Rao et al., 2013). The following genes were selected here for analyses based on their established roles in neuroinflammation and/or neuroendocrine functions: *c-fos* (a neuronal marker of activation/arousal, often upregulated in stress), genes of pro-inflammatory cytokines interferon INF-γ, tumor necrosis factor TNF-α and interleukins (IL) IL-1β (often upregulated by stress), genes of anti-inflammatory cytokines IL-10, IL-4, neurotrophin brain-derived neurotrophic factor (BNDF), selected HPI axis-related genes encoding Steroidogenic acute regulatory protein (StAr), Pro-opiomelanocortin (POMC), brain glucocorticoid receptor (BGR), and stress hormone corticotropin-releasing factor (CRF). The primers used for these genes are presented in Table 2.

### Statistical analysis

Data were analyzed using the unpaired *t*-test or Mann–Whitney *U*-test, depending on data normality, as assessed by the Kolmogorov–Smirnov test, and homogeneity of variance, determined using the Hartley’s test. *p* was set at < 0.05 for all tests.

## Results

**Figure 1:**
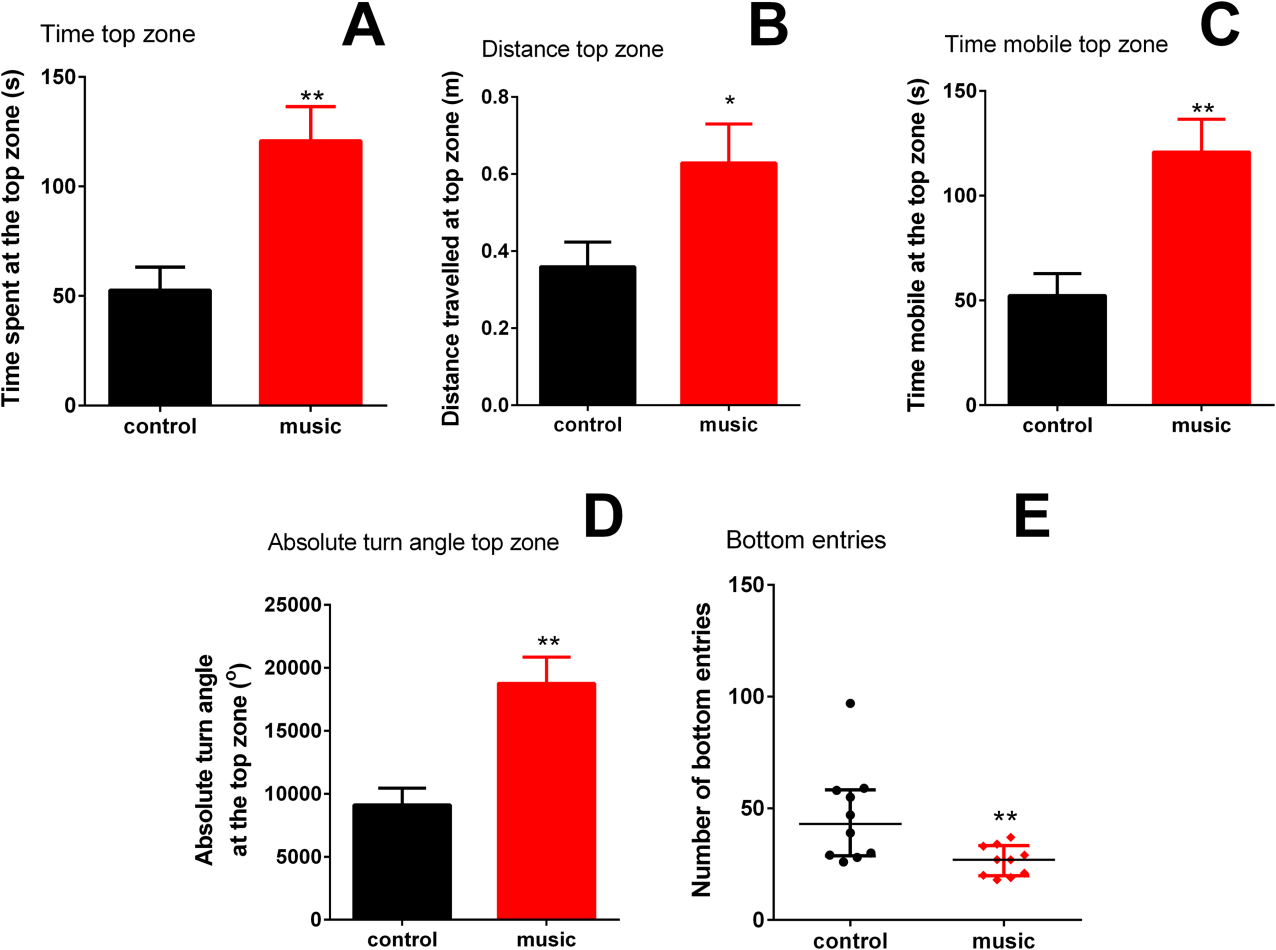
Behavioral performance of zebrafish in the novel tank test (NTT) following daily exposure to auditory enrichment (Vivaldi’s music) for 15 days. Data from top zone ((A) time spent at the top zone; (B) distance travelled at the top zone; (C) time mobile at the top zone and (D) absolute turn angle at the top zone) are expressed as mean ± S.E.M. and analyzed by unpaired *t*-test. Data from the NTT bottom zone ((E) number of the bottom entries) are expressed as median ± interquartile range and analyzed by Mann–Whitney *U*-test. *p < 0.05; **p < 0.01 vs. unexposed control (*n* = 10).

Overall, fish exposed to music clearly preferred the top NTT zone (*p* = 0.002) and spent significantly less time at the tank bottom (*p* = 0.0116). In the top, they also travelled longer distance (*p* = 0.0370), spent more time moving (mobile) (*p* = 0.0019), they showed higher absolute turn angle (*p* = 0.0011), compared to unexposed controls. In the bottom zone of the NTT, the number of entries into this area (*p* = 0.0095) was significantly lower than controls (Fig. 1), collectively suggesting an anxiolytic-like behavioral profile evoked by music exposure in the experimental group.

**Figure 2:**
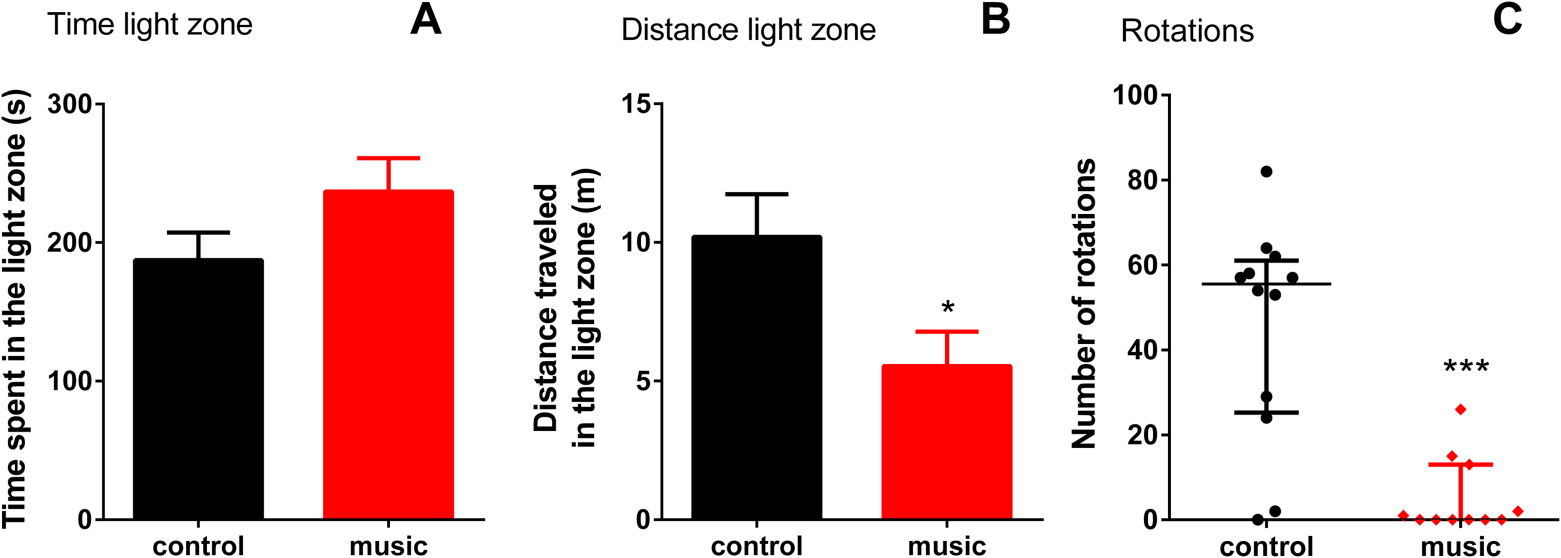
Behavioral performance of zebrafish in the light-dark test (LDT) following daily exposure to auditory enrichment (Vivaldi’s music) for 15 days. Data from time spent (A) and distance travelled in light zone (B) were expressed as mean ± S.E.M. and analyzed by unpaired *t*-test. Number of rotations in the light zone (C) were expressed as median ± interquartile range and analyzed by Mann–Whitney test. *p < 0.05; ***p < 0.001 vs. unexposed control (*n* = 12).

In the LDT, there were no differences between the groups in time spent in light (*p* = 0.1267), although fish exposed to music appeared calmer as they travelled shorter distance in the light zone (*p* = 0.0299) and showed fewer rotations (*p* = 0.0004, Fig. 2).

**Figure 3:**
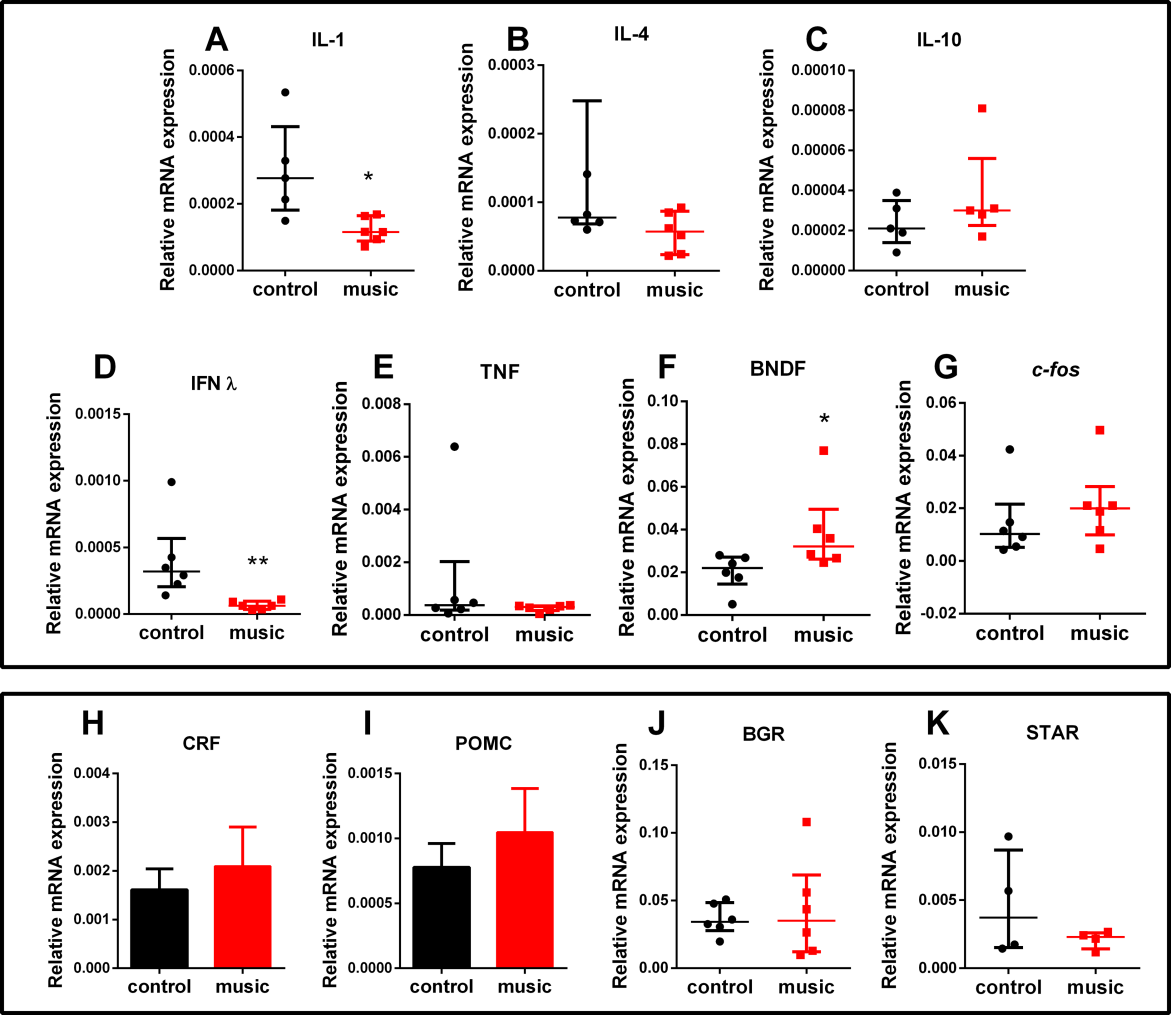
Relative mRNA expression of immune and HPI axis-related brain genes in zebrafish exposed daily to auditory enrichment (Vivaldi’s music) for 15 days. (A) IL-1; (B) IL-4; (C) IL-10; (D) IFNλ; (E) TNF; (F) BDNF; (G) *c-fos*; (H) CRF; (I) POMC; (J)BGR and (K) StAR. Parametric data for *POMC* and *CRF* expression are expressed as mean ± S.E.M. and analyzed by unpaired *t*-test. Data for other genes are non-parametric and expressed as median ± interquartile range, analyzed by Mann–Whitney test. *p < 0.05; **p < 0.01 vs. unexposed control (*n* = 6). Abbreviations of the genes are as in Table 2.

The CNS gene expression results are presented in Fig. 3. Overall, affecting the group of immune genes, auditory enrichment decreased the expression of pro-inflammatory IL IL-1β (*p* = 0.0173) and INF-γ (*p* = 0.0022), but did not affect other cytokines IL-4 (*p* = 0.1797, NS), IL-10 (*p* = 0.3016, NS), and TNF-α (*p* = 0.4740, NS). Additionally, music exposure elevated the expression of *BNDF* (*p* = 0.0260), but not *c-fos* (*p* = 0.2229, NS) or selected HPI axis-related genes *StAr* (*p* = 0.6571, NS), *POMC* (*p* = 0.4961, NS), *BGR* (*p* = 0.8983, NS), and *CRF* (*p* = 0.6063, NS).

Finally, the trunk cortisol levels did not differ between the groups (*p* = 0.5371, *n* = 8), with fish exposed to music yielding 11.88 ± 1.41 vs. control 10.25 ± 2.1 ng/g tissue.

## Discussion

Mounting evidence supports the role of various types of environmental enrichment in zebrafish models (Schroeder et al., 2014; Collymore, Tolwani & Rasmussen, 2015; Manuel et al., 2015). To the best of our knowledge, the present study is the first report examining the role of auditory enrichment, such as 15-day repeated classical (Vivaldi) music exposure, on zebrafish behavior and physiology. In the NTT, fish chronically exposed to this type of auditory enrichment were less anxious and most active, compared to unexposed control group (Fig. 1). In addition, the exposed group showed no overt stress responses (vs. control) in whole-body cortisol assay and unaltered expression of CNS genes related to stress response (Fig. 3B). The baseline behavioral response of control fish tested in the NTT (e.g., spending more time in the bottom, Fig. 1) resembled other studies using this model (Egan et al., 2009) and was generally expected, since the test novelty is a stressful factor for zebrafish (Kysil et al., 2017). In contrast, fish exposed to specific auditory enrichment (Vivaldi’s music) used here were clearly less anxious even facing the NTT novelty, strikingly paralleling “anxiolytic” effects of Mozart’s music in humans (Rickard, Toukhsati & Field, 2005) and rodents (Alworth & Buerkle, 2013). While the two composers clearly differ in their styles, the overall high level of auditory harmony of their music is widely recognized (Mammarella, Fairfield & Cornoldi, 2013) and likely contributed to the similar behavioral effects observed here. However, comparing present auditory enrichment with other types of music and/or non-music sound stimulation in zebrafish was beyond the scope of this study.

Interestingly, although the LDT results somewhat differed from the NTT findings (Fig. 2) described above, the fact that music-exposed fish were less active than controls suggests that they were also generally calmer in the light zone. This response may also reflect the fact that LDT has a limited ability to detect anxiolytic responses, compared to zebrafish NTT (Kysil et al., 2017), and the LDT inherent limitation as a model since substantial portion of fish behaviors in the dark section of the apparatus remained unaccounted for in this test.

Furthermore, specific type of auditory enrichment used here also affected the immune genes expression in zebrafish vs. unexposed controls (Fig. 3A), similar to music effects reported earlier in rodents (Lu et al., 2010; Uchiyama et al., 2012). Here, fish exposed to auditory enrichment showed lower expression of some pro-inflammatory genes (IL-1β and IFN-γ), but without affecting anti-inflammatory genes *IL-10* and *IL-4* (Fig. 3A). Notably, both Vivaldi’s and Mozart’s music seem to positively modulate neuronal activation at hippocampal and enhance spatial cognition ability in rodents, based on their up-regulation of *BDNF* (Xing et al., 2016), which can also contribute to anxiolytic-like profile observed here in zebrafish (Fig. 2). In contrast, we did not observe the effect of music on *c-fos* expression in the brain. Although this early proto-oncogene is a well-established marker of stress reactivity in the brain (Bouwknecht et al., 2007) and can be upregulated by noise stress in rats (Babb et al., 2013), the baseline differences in stress reactivity in music-exposed vs. control fish may not be robust here, especially since zebrafish trunk cortisol levels also remained unaltered. Overall, the observed behavioral phenotypes (Figs. 1 and 2) suggest that auditory stimulation may have an anxiolytic-like effect in zebrafish, compared to unexposed controls. Furthermore, our method of auditory stimulus presentation differs from that of other groups (Papoutsoglou et al., 2007; Imanpoor, Enayat Gholampour & Zolfaghari, 2011) who introduced hydrophones directly into the aquatic environments. While the latter method requires an expensive experimental equipment, our easier and cheaper method (utilizing a simple MP3 player) can be advantageous from the practical point of view.

One limitation of our study is that it did not measure the intensity level of the sound signal coming into the fish tank water. However, this technical aspect does not negate the overall relevance of our results, for the first time revealing the role of repeated musical auditory environmental enrichment in zebrafish. As already mentioned, the 65–75 dB sound range in the laboratory room was chosen as pleasing to humans (Brookhouser, 1994), but it remains unclear how zebrafish perceive it. Testing more loud sounds (e.g., using the same music but at different loudness levels) may also be interesting, and can be performed in subsequent follow-up studies. However, such studies are rather problematic in the research facility, and are unlikely feasible or practical for other laboratories as an auditory enrichment, since it would create a major discomfort to researchers and technicians, and may also distress all species of laboratory animals.

Nevertheless, we note that fishes can discriminate sound intensity and frequency, as well as localize the sound source and analyze auditory signal spectra (Fay & Popper, 2000). Several questions remain open for future studies in zebrafish models. For example, would other composers and even music types evoke similar, or different, behavioral profiles, in fish? Will these responses be similar with those of another species, like rats (Otsuka, Yanagi & Watanabe, 2009) or birds (Watanabe & Nemoto, 1998)? And, if there were a difference, to what extent the behavioral outcome recorded would depend on baseline housing factors, such as background noise present in specific laboratory environments, as well as whether inter-laboratory differences in such auditory backgrounds may affect the observed behavioral outcomes? Indeed, the effect of other husbandry factors, such as lighting, have been reported to affect stress responsivity in rodents (Bouwknecht et al., 2007). Thus, the possibility of similar effects of “sound background” in rodent or fish models remains unclear, and merits further scrutiny in zebrafish tests.

Likewise, in addition to *c-fos* and cortisol assays, other hormonal and molecular biomarkers, such as neurochemical alterations and/or stress-related peripheral or central cytokines, may be examined in-depth in the follow-up studies. The patterns of brain gene expression and epigenetic modifications may also be examined in such studies, including recently developed methods such as differential gene expression analyses (Gutha et al., 2018). Furthermore, music exposure for a longer period of time (e.g., 5–10 weeks) and/or more frequently (e.g., 3–4 h twice a day) may be utilized in future studies, to more fully characterize long-term auditory enrichment effects in zebrafish. Clearly, the latter protocols may be more relevant to prolonged sound exposure in laboratory housing environments, providing important novel insights into zebrafish husbandry and their phenomics. Again, using additional control groups, including exposure to white noise as well as other musical and non-musical sounds, can be a useful future line of research in this model. Finally, combining behavioral and physiological analyses in such studies with additional neuromorphological assays relevant to brain plasticity, such as examining synaptic density, neuronal arborization, and/or dendritic spines, may also be warranted in zebrafish and other aquatic species.

## Conclusion

In summary, zebrafish exposed to specific type of auditory enrichment (twice daily exposure to Vivaldi’s music for two weeks) were less anxious and more active, compared to their unexposed control counterparts. The exposed fish also showed upregulated pro-inflammatory genes *IL-1β* and *INF*γ, as well as the neurotrophin *BNDF* gene in the brain. Taken together, these findings suggest that the used auditory enrichment in zebrafish may be a potential factor modulating their behavioral and physiological responses. In essence, we report that twice daily exposure to continuous 65–75 dB sounds may provide benefits over the ongoing background noise of equipment in the laboratory setting. From the practical standpoint, these results support using musical environmental enrichment in zebrafish, similar to auditory enrichment currently used in rodents Moreover, it has still not been established that the melodic content of the music is responsible for the effects reported here, although some studies show that animals react differently to music and other sounds, such as static (Kettelkamp-Ladd, 1993). For example, it has been repeatedly demonstrated that non-musical sound alone may have a beneficial effect on animals (Robbins & Margulis, 2014; Robbins & Margulis, 2016; Pysanenko et al., 2018), and therefore our conclusions are limited to auditory enrichment in general, rather than to music more specifically.

## Supplemental Information

10.7717/peerj.5162/supp-1 Supplemental Information 1 Raw data and statistics.

## Additional Information and Declarations

### Data Availability

The following information was supplied regarding data availability: The raw data and statistics are included in the Supplemental Dataset Files.
