# Supplementary material for: The effects of auditory enrichment on zebrafish behavior and physiology
Source: PeerJ. 2018 Jul 23;6:e5162. doi: 10.7717/peerj.5162 (PMC6061163; doi:10.7717/peerj.5162)
Supplement: Supplemental Information 1 [file peerj-06-5162-s001.docx]

Barcellos et al.

Zebrafish chronically exposed to Vivaldi music are calmer and less anxious: potential implications for environmental enrichment and neurophenomic research in aquatic models

**Raw data and statistics**

**Figure 1.**

1. Time spent in the top zone

| control | music |
| --- | --- |
| 34.2 | 142.0 |
| 7.4 | 83.1 |
| 94.1 | 117.4 |
| 36.1 | 241.5 |
| 53.7 | 111.6 |
| 101.0 | 54.8 |
| 95.0 | 133.2 |
| 50.0 | 105.9 |
| 37.6 | 126.1 |
| 16.6 | 92.1 |

| Table Analyzed | time in top zone |
| --- | --- |
|  |  |
| Column B | music |
| vs. | vs. |
| Column A | control |
|  |  |
| Unpaired t test |  |
| P value | 0.0020 |
| P value summary | ** |
| Significantly different? (P < 0.05) | Yes |
| One- or two-tailed P value? | Two-tailed |
| t. df | t=3.608 df=18 |
|  |  |
| How big is the difference? |  |
| Mean ± SEM of column A | 52.57 ± 10.56 N=10 |
| Mean ± SEM of column B | 120.8 ± 15.68 N=10 |
| Difference between means | 68.20 ± 18.90 |
| 95% confidence interval | 28.48 to 107.9 |
| R square | 0.4196 |
|  |  |
| F test to compare variances |  |
| F.DFn. Dfd | 2.202. 9. 9 |
| P value | 0.2553 |
| P value summary | ns |
| Significantly different? (P < 0.05) | No |

1. Distance top zone

| control | music |
| --- | --- |
| 0.284 | 0.690 |
| 0.062 | 0.578 |
| 0.532 | 0.689 |
| 0.269 | 1.470 |
| 0.356 | 0.526 |
| 0.461 | 0.274 |
| 0.616 | 0.459 |
| 0.655 | 0.588 |
| 0.248 | 0.548 |
| 0.110 | 0.466 |

| Table Analyzed | distance traveled at top zone |
| --- | --- |
|  |  |
| Column B | music |
| vs. | vs. |
| Column A | control |
|  |  |
| Unpaired t test |  |
| P value | 0.0370 |
| P value summary | * |
| Significantly different? (P < 0.05) | Yes |
| One- or two-tailed P value? | Two-tailed |
| t. df | t=2.253 df=18 |
|  |  |
| How big is the difference? |  |
| Mean ± SEM of column A | 0.3593 ± 0.06414 N=10 |
| Mean ± SEM of column B | 0.6288 ± 0.1010 N=10 |
| Difference between means | 0.2695 ± 0.1196 |
| 95% confidence interval | 0.01819 to 0.5208 |
| R square | 0.2200 |
|  |  |
| F test to compare variances |  |
| F.DFn. Dfd | 2.478. 9. 9 |
| P value | 0.1926 |
| P value summary | ns |
| Significantly different? (P < 0.05) | No |

1. Time mobile top zone

| control | music |
| --- | --- |
| 34.2 | 142.0 |
| 7.4 | 83.1 |
| 94.1 | 117.4 |
| 36.1 | 241.5 |
| 53.7 | 111.6 |
| 101.0 | 54.8 |
| 92.8 | 133.2 |
| 50.0 | 105.9 |
| 37.6 | 126.1 |
| 16.6 | 92.1 |

| Table Analyzed | time mobile at top zone |
| --- | --- |
|  |  |
| Column B | music |
| vs. | vs. |
| Column A | control |
|  |  |
| Unpaired t test |  |
| P value | 0.0019 |
| P value summary | ** |
| Significantly different? (P < 0.05) | Yes |
| One- or two-tailed P value? | Two-tailed |
| t. df | t=3.630 df=18 |
|  |  |
| How big is the difference? |  |
| Mean ± SEM of column A | 52.35 ± 10.47 N=10 |
| Mean ± SEM of column B | 120.8 ± 15.68 N=10 |
| Difference between means | 68.42 ± 18.85 |
| 95% confidence interval | 28.82 to 108.0 |
| R square | 0.4226 |
|  |  |
| F test to compare variances |  |
| F.DFn. Dfd | 2.243. 9. 9 |
| P value | 0.2447 |
| P value summary | ns |
| Significantly different? (P < 0.05) | No |

1. Absolute turn angle top zone

| control | music |
| --- | --- |
| 8840. | 25224. |
| 1290. | 13848. |
| 12954. | 18732. |
| 7457. | 32659. |
| 11437. | 15826. |
| 11677. | 10477. |
| 15422. | 15533. |
| 10971. | 19040. |
| 6575. | 22749. |
| 4498. | 13509. |

| Table Analyzed | absolute turn angle into top zone |
| --- | --- |
|  |  |
| Column B | music |
| vs. | vs. |
| Column A | control |
|  |  |
| Unpaired t test |  |
| P value | 0.0011 |
| P value summary | ** |
| Significantly different? (P < 0.05) | Yes |
| One- or two-tailed P value? | Two-tailed |
| t. df | t=3.895 df=18 |
|  |  |
| How big is the difference? |  |
| Mean ± SEM of column A | 9112 ± 1341 N=10 |
| Mean ± SEM of column B | 18760 ± 2082 N=10 |
| Difference between means | 9648 ± 2477 |
| 95% confidence interval | 4444 to 14851 |
| R square | 0.4574 |
|  |  |
| F test to compare variances |  |
| F.DFn. Dfd | 2.413. 9. 9 |
| P value | 0.2056 |
| P value summary | ns |
| Significantly different? (P < 0.05) | No |

1. Bottom entries

| control | music |
| --- | --- |
| 55 | 37 |
| 26 | 29 |
| 29 | 27 |
| 59 | 18 |
| 47 | 19 |
| 30 | 27 |
| 58 | 21 |
| 97 | 34 |
| 39 | 20 |
| 28 | 33 |

| Table Analyzed | bottom entries |
| --- | --- |
|  |  |
| Column B | music |
| vs. | vs. |
| Column A | control |
|  |  |
| Mann Whitney test |  |
| P value | 0.0095 |
| Exact or approximate P value? | Exact |
| P value summary | ** |
| Significantly different? (P < 0.05) | Yes |
| One- or two-tailed P value? | Two-tailed |
| Sum of ranks in column A.B | 138.5 . 71.50 |
| Mann-Whitney U | 16.50 |
|  |  |
| Difference between medians |  |
| Median of column A | 43.00 |
| Median of column B | 27.00 |
| Difference: Actual | -16.00 |
| Difference: Hodges-Lehmann | -18.00 |

**Figure 2.**

1. Time light zone

| control | music |
| --- | --- |
| 296 | 339 |
| 179 | 214 |
| 233 | 166 |
| 84 | 266 |
| 278 | 216 |
| 213 | 130 |
| 192 | 359 |
| 229 | 290 |
| 182 | 153 |
| 86 | 183 |
| 167 | 166 |
| 108 | 360 |

| Table Analyzed | time into light zone |
| --- | --- |
|  |  |
| Column B | music |
| vs. | vs. |
| Column A | control |
|  |  |
| Unpaired t test |  |
| P value | 0.1267 |
| P value summary | ns |
| Significantly different? (P < 0.05) | No |
| One- or two-tailed P value? | Two-tailed |
| t. df | t=1.587 df=22 |
|  |  |
| How big is the difference? |  |
| Mean ± SEM of column A | 187.3 ± 19.90 N=12 |
| Mean ± SEM of column B | 236.8 ± 24.08 N=12 |
| Difference between means | 49.58 ± 31.24 |
| 95% confidence interval | -15.20 to 114.4 |
| R square | 0.1027 |
|  |  |
| F test to compare variances |  |
| F.DFn. Dfd | 1.464. 11. 11 |
| P value | 0.5378 |
| P value summary | ns |
| Significantly different? (P < 0.05) | No |

1. Distance light zone

| control | music |
| --- | --- |
| 14.816 | 3.236 |
| 10.719 | 0.000 |
| 10.990 | 5.861 |
| 0.417 | 14.708 |
| 15.506 | 6.362 |
| 14.865 | 7.521 |
| 12.709 | 2.104 |
| 16.082 | 8.254 |
| 2.491 | 5.436 |
| 5.378 | 6.810 |
| 12.262 | 0.680 |
| 6.143 |  |

| Table Analyzed | distance traveled at light zone |
| --- | --- |
|  |  |
| Column B | music |
| vs. | vs. |
| Column A | control |
|  |  |
| Unpaired t test |  |
| P value | 0.0299 |
| P value summary | * |
| Significantly different? (P < 0.05) | Yes |
| One- or two-tailed P value? | Two-tailed |
| t. df | t=2.330 df=21 |
|  |  |
| How big is the difference? |  |
| Mean ± SEM of column A | 10.20 ± 1.538 N=12 |
| Mean ± SEM of column B | 5.543 ± 1.240 N=11 |
| Difference between means | -4.655 ± 1.998 |
| 95% confidence interval | -8.811 to -0.4995 |
| R square | 0.2054 |
|  |  |
| F test to compare variances |  |
| F.DFn. Dfd | 1.677. 11. 10 |
| P value | 0.4239 |
| P value summary | ns |
| Significantly different? (P < 0.05) | No |

1. rotations

| control | music |
| --- | --- |
| 82 | 0 |
| 53 | 0 |
| 57 | 2 |
| 0 | 26 |
| 58 | 13 |
| 62 | 15 |
| 54 | 1 |
| 64 | 0 |
| 2 | 0 |
| 24 | 0 |
| 57 | 0 |
| 29 |  |

| Table Analyzed | rotation |
| --- | --- |
|  |  |
| Column B | music |
| vs. | vs. |
| Column A | control |
|  |  |
| Mann Whitney test |  |
| P value | 0.0004 |
| Exact or approximate P value? | Exact |
| P value summary | *** |
| Significantly different? (P < 0.05) | Yes |
| One- or two-tailed P value? | Two-tailed |
| Sum of ranks in column A.B | 197.5 . 78.50 |
| Mann-Whitney U | 12.50 |
|  |  |
| Difference between medians |  |
| Median of column A | 55.50 |
| Median of column B | 0.0 |
| Difference: Actual | -55.50 |
| Difference: Hodges-Lehmann | -51.50 |

**Figure 3.**

IL-1

| control | music |
| --- | --- |
| 0.000329 | 0.000115 |
| 0.000213 | 0.000072 |
| 0.000534 | 0.000116 |
| 0.000277 | 0.000094 |
| 0.000149 | 0.000168 |
|  | 0.000163 |

| Table Analyzed | IL-1 |
| --- | --- |
|  |  |
| Column B | music |
| vs. | vs. |
| Column A | control |
|  |  |
| Mann Whitney test |  |
| P value | 0.0173 |
| Exact or approximate P value? | Exact |
| P value summary | * |
| Significantly different? (P < 0.05) | Yes |
| One- or two-tailed P value? | Two-tailed |
| Sum of ranks in column A.B | 43.00 . 23.00 |
| Mann-Whitney U | 2.000 |
|  |  |
| Difference between medians |  |
| Median of column A | 0.000277 |
| Median of column B | 0.0001155 |
| Difference: Actual | -0.0001615 |
| Difference: Hodges-Lehmann | -0.000161 |

IL-4

| control | music |
| --- | --- |
| 0.000082 | 0.000062 |
| 0.000071 | 0.000024 |
| 0.000569 | 0.000052 |
| 0.000060 | 0.000022 |
| 0.000073 | 0.000085 |
| 0.000141 | 0.000092 |

| Table Analyzed | IL4 |
| --- | --- |
|  |  |
| Column B | music |
| vs. | vs. |
| Column A | control |
|  |  |
| Mann Whitney test |  |
| P value | 0.1797 |
| Exact or approximate P value? | Exact |
| P value summary | ns |
| Significantly different? (P < 0.05) | No |
| One- or two-tailed P value? | Two-tailed |
| Sum of ranks in column A.B | 48.00 . 30.00 |
| Mann-Whitney U | 9.000 |
|  |  |
| Difference between medians |  |
| Median of column A | 7.750e-005 |
| Median of column B | 5.700e-005 |
| Difference: Actual | -2.050e-005 |
| Difference: Hodges-Lehmann | -4.250e-005 |

IL-10

| control | music |
| --- | --- |
| 0.000019 | 0.000030 |
| 0.000009 | 0.000028 |
| 0.000031 | 0.000031 |
| 0.000021 | 0.000017 |
| 0.000039 | 0.000081 |

| Table Analyzed | IL-10 |
| --- | --- |
|  |  |
| Column B | music |
| vs. | vs. |
| Column A | control |
|  |  |
| Unpaired t test |  |
| P value | 0.3016 |
| P value summary | ns |
| Significantly different? (P < 0.05) | No |
| One- or two-tailed P value? | Two-tailed |
| t. df | t=1.104 df=8 |
|  |  |
| How big is the difference? |  |
| Mean ± SEM of column A | 2.380e-005 ± 5.161e-006 N=5 |
| Mean ± SEM of column B | 3.740e-005 ± 1.118e-005 N=5 |
| Difference between means | 1.360e-005 ± 1.232e-005 |
| 95% confidence interval | -1.480e-005 to 4.200e-005 |
| R square | 0.1323 |
|  |  |
| F test to compare variances |  |
| F.DFn. Dfd | 4.694. 4. 4 |
| P value | 0.1634 |
| P value summary | ns |
| Significantly different? (P < 0.05) | No |

IFNƛ

| control | music |
| --- | --- |
| 0.000425 | 0.000063 |
| 0.000226 | 0.000062 |
| 0.000290 | 0.000036 |
| 0.000140 | 0.000092 |
| 0.000990 | 0.000109 |
| 0.000348 | 0.000030 |

| Table Analyzed | INF gama |
| --- | --- |
|  |  |
| Column B | music |
| vs. | vs. |
| Column A | control |
|  |  |
| Mann Whitney test |  |
| P value | 0.0022 |
| Exact or approximate P value? | Exact |
| P value summary | ** |
| Significantly different? (P < 0.05) | Yes |
| One- or two-tailed P value? | Two-tailed |
| Sum of ranks in column A.B | 57.00 . 21.00 |
| Mann-Whitney U | 0.0 |
|  |  |
| Difference between medians |  |
| Median of column A | 0.000319 |
| Median of column B | 6.250e-005 |
| Difference: Actual | -0.0002565 |
| Difference: Hodges-Lehmann | -0.000255 |

TNF

| control | music |
| --- | --- |
| 0.000465 | 0.000041 |
| 0.000225 | 0.000275 |
| 0.000569 | 0.000210 |
| 0.000062 | 0.000370 |
| 0.006390 | 0.000329 |
| 0.000273 | 0.000341 |

| Table Analyzed | TNF |
| --- | --- |
|  |  |
| Column B | music |
| vs. | vs. |
| Column A | control |
|  |  |
| Mann Whitney test |  |
| P value | 0.4740 |
| Exact or approximate P value? | Exact |
| P value summary | ns |
| Significantly different? (P < 0.05) | No |
| One- or two-tailed P value? | Two-tailed |
| Sum of ranks in column A.B | 44.00 . 34.00 |
| Mann-Whitney U | 13.00 |
|  |  |
| Difference between medians |  |
| Median of column A | 0.000369 |
| Median of column B | 0.000302 |
| Difference: Actual | -6.700e-005 |
| Difference: Hodges-Lehmann | -0.00013 |

BDNF

| control | music |
| --- | --- |
| 0.026830 | 0.035897 |
| 0.024014 | 0.040386 |
| 0.019915 | 0.076947 |
| 0.027970 | 0.026645 |
| 0.005013 | 0.028360 |
| 0.017579 | 0.024518 |

c-FOS

| control | music |
| --- | --- |
| 0.009163 | 0.021051 |
| 0.042394 | 0.018841 |
| 0.014680 | 0.049721 |
| 0.005486 | 0.021051 |
| 0.004304 | 0.011679 |
| 0.011438 | 0.004645 |

| Table Analyzed | BNDF |
| --- | --- |
|  |  |
| Column B | music |
| vs. | vs. |
| Column A | control |
|  |  |
| Mann Whitney test |  |
| P value | 0.0260 |
| Exact or approximate P value? | Exact |
| P value summary | * |
| Significantly different? (P < 0.05) | Yes |
| One- or two-tailed P value? | Two-tailed |
| Sum of ranks in column A.B | 25.00 . 53.00 |
| Mann-Whitney U | 4.000 |
|  |  |
| Difference between medians |  |
| Median of column A | 0.02196 |
| Median of column B | 0.03213 |
| Difference: Actual | 0.01016 |
| Difference: Hodges-Lehmann | 0.01215 |
| Table Analyzed | cFOS |
|  |  |
| Column B | music |
| vs. | vs. |
| Column A | control |
|  |  |
| Mann Whitney test |  |
| P value | 0.2229 |
| Exact or approximate P value? | Exact |
| P value summary | ns |
| Significantly different? (P < 0.05) | No |
| One- or two-tailed P value? | Two-tailed |
| Sum of ranks in column A.B | 31.00 . 47.00 |
| Mann-Whitney U | 10.00 |
|  |  |
| Difference between medians |  |
| Median of column A | 0.01030 |
| Median of column B | 0.01995 |
| Difference: Actual | 0.009646 |
| Difference: Hodges-Lehmann | 0.007351 |

(B)

CRF

| control | music |
| --- | --- |
| 0.002762 | 0.005448 |
| 0.002920 | 0.003521 |
| 0.001047 | 0.001410 |
| 0.001785 | 0.000499 |
| 0.000422 | 0.000990 |
| 0.000740 | 0.000720 |

| Table Analyzed | CRF |
| --- | --- |
|  |  |
| Column B | music |
| vs. | vs. |
| Column A | control |
|  |  |
| Unpaired t test |  |
| P value | 0.6063 |
| P value summary | ns |
| Significantly different? (P < 0.05) | No |
| One- or two-tailed P value? | Two-tailed |
| t. df | t=0.5320 df=10 |
|  |  |
| How big is the difference? |  |
| Mean ± SEM of column A | 0.001613 ± 0.0004305 N=6 |
| Mean ± SEM of column B | 0.002098 ± 0.0008042 N=6 |
| Difference between means | 0.0004853 ± 0.0009122 |
| 95% confidence interval | -0.001547 to 0.002518 |
| R square | 0.02753 |
|  |  |
| F test to compare variances |  |
| F.DFn. Dfd | 3.491. 5. 5 |
| P value | 0.1963 |
| P value summary | ns |
| Significantly different? (P < 0.05) | No |

POMC

| control | music |
| --- | --- |
| 0.001642 | 0.001609 |
| 0.000577 | 0.000720 |
| 0.000557 | 0.000226 |
| 0.000378 | 0.000386 |
| 0.000715 | 0.000943 |
| 0.000799 | 0.002405 |

| Table Analyzed | POMC |
| --- | --- |
|  |  |
| Column B | music |
| vs. | vs. |
| Column A | control |
|  |  |
| Unpaired t test |  |
| P value | 0.4961 |
| P value summary | ns |
| Significantly different? (P < 0.05) | No |
| One- or two-tailed P value? | Two-tailed |
| t. df | t=0.7063 df=10 |
|  |  |
| How big is the difference? |  |
| Mean ± SEM of column A | 0.000778 ± 0.0001826 N=6 |
| Mean ± SEM of column B | 0.001048 ± 0.0003361 N=6 |
| Difference between means | 0.0002702 ± 0.0003825 |
| 95% confidence interval | -0.0005821 to 0.001122 |
| R square | 0.04752 |
|  |  |
| F test to compare variances |  |
| F.DFn. Dfd | 3.388. 5. 5 |
| P value | 0.2066 |
| P value summary | ns |
| Significantly different? (P < 0.05) | No |

BGR

| control | music |
| --- | --- |
| 0.030607 | 0.055939 |
| 0.035897 | 0.012780 |
| 0.047696 | 0.026461 |
| 0.019641 | 0.009685 |
| 0.032577 | 0.043586 |
| 0.050766 | 0.108067 |

| Table Analyzed | BGR |
| --- | --- |
|  |  |
| Column B | music |
| vs. | vs. |
| Column A | control |
|  |  |
| Mann Whitney test |  |
| P value | 0.8983 |
| Exact or approximate P value? | Exact |
| P value summary | ns |
| Significantly different? (P < 0.05) | No |
| One- or two-tailed P value? | Two-tailed |
| Sum of ranks in column A.B | 40.00 . 38.00 |
| Mann-Whitney U | 17.00 |
|  |  |
| Difference between medians |  |
| Median of column A | 0.03424 |
| Median of column B | 0.03502 |
| Difference: Actual | 0.0007865 |
| Difference: Hodges-Lehmann | -0.004128 |

STAR

| control | music |
| --- | --- |
| 0.001440 | 0.002668 |
| 0.005680 | 0.002421 |
| 0.009685 | 0.002167 |
| 0.001748 | 0.001169 |

| Mann Whitney test |  |
| --- | --- |
| P value | 0.6571 |
| Exact or approximate P value? | Exact |
| P value summary | ns |
| Significantly different? (P < 0.05) | No |
| One- or two-tailed P value? | Two-tailed |
| Sum of ranks in column A.B | 20.00 . 16.00 |
| Mann-Whitney U | 6.000 |
|  |  |
| Difference between medians |  |
| Median of column A | 0.003714 |
| Median of column B | 0.002294 |
| Difference: Actual | -0.00142 |
| Difference: Hodges-Lehmann | -0.001796 |

Cortisol (data nos show)

| control | music |
| --- | --- |
| 1.6 | 1.6 |
| 1.9 | 0.4 |
| 2.7 | 2.2 |
| 1.0 | 2.0 |
| 2.5 | 2.4 |
| 3.0 | 4.6 |
| 3.0 | 1.2 |
| 3.3 | 2.0 |

| Unpaired t test |  |
| --- | --- |
| P value | 0,5371 |
| P value summary | ns |
| Significantly different? (P < 0.05) | No |
| One- or two-tailed P value? | Two-tailed |
| t, df | t=0,6327 df=14 |
|  |  |
| How big is the difference? |  |
| Mean ± SEM of column A | 2,375 ± 0,2827 N=8 |
| Mean ± SEM of column B | 2,050 ± 0,4289 N=8 |
| Difference between means | -0,3250 ± 0,5137 |
| 95% confidence interval | -1,427 to 0,7767 |
| R square | 0,02780 |
|  |  |
| F test to compare variances |  |
| F,DFn, Dfd | 2,302, 7, 7 |
| P value | 0,2937 |
| P value summary | ns |
| Significantly different? (P < 0.05) | No |
